# Multistep Signaling in Nature: A Close-Up of *Geobacter* Chemotaxis Sensing

**DOI:** 10.3390/ijms22169034

**Published:** 2021-08-21

**Authors:** Marta A. Silva, Carlos A. Salgueiro

**Affiliations:** 1 Associate Laboratory i4HB—Institute for Health and Bioeconomy, NOVA School of Science and Technology, NOVA University Lisbon, 2819-516 Caparica, Portugal; mafs@fct.unl.pt; 2 UCIBIO—Applied Molecular Biosciences Unit, Department of Chemistry, NOVA School of Science and Technology, NOVA University Lisbon, 2819-516 Caparica, Portugal

**Keywords:** two-component system, methyl-accepting chemotaxis proteins, signal transduction, redox-sensing, *Geobacter*, *c*-type heme sensor domains

## Abstract

Environmental changes trigger the continuous adaptation of bacteria to ensure their survival. This is possible through a variety of signal transduction pathways involving chemoreceptors known as methyl-accepting chemotaxis proteins (MCP) that allow the microorganisms to redirect their mobility towards favorable environments. MCP are two-component regulatory (or signal transduction) systems (TCS) formed by a sensor and a response regulator domain. These domains synchronize transient protein phosphorylation and dephosphorylation events to convert the stimuli into an appropriate cellular response. In this review, the variability of TCS domains and the most common signaling mechanisms are highlighted. This is followed by the description of the overall cellular topology, classification and mechanisms of MCP. Finally, the structural and functional properties of a new family of MCP found in *Geobacter sulfurreducens* are revisited. This bacterium has a diverse repertoire of chemosensory systems, which represents a striking example of a survival mechanism in challenging environments. Two *G. sulfurreducens* MCP—GSU0582 and GSU0935—are members of a new family of chemotaxis sensor proteins containing a periplasmic PAS-like sensor domain with a *c*-type heme. Interestingly, the cellular location of this domain opens new routes to the understanding of the redox potential sensing signaling transduction pathways.

## 1. Introduction

Sensing environmental signals by chemoreceptors and the concomitant triggering of efficient adaptation responses, such as chemoattraction or chemorepellence, are key features of cell survival. Chemoreceptors constitute a highly diverse protein superfamily that differs in topology, cellular location, dimension, domain composition, function or even in the response mechanism. This review focuses on a particular group of chemoreceptors known as two-component systems (TCS). TCS are the most abundant multistep signaling pathways in Nature, and despite their crucial role in the cellular signaling strategies, the physiological signals that modulate their action are not precisely understood. TCS are found in organisms of all domains (Bacteria, Archaea, and Eukarya); however, their abundance in each domain differs substantially [1]. They are abundant in most Bacteria and constitute about ∼1% of the encoded proteins. For example, the *Escherichia coli* (*E. coli*) genome encodes for 62 TCS proteins that are involved in the regulation of several processes, namely chemotaxis, osmoregulation, cellular metabolism and biological transport [2]. In addition to the common intrinsic regulatory functions, these systems are also important in pathogenic bacteria to control expression of toxins and key proteins for their pathogenesis [3,4]. In Archaea and Eukarya, the TCS pathways constitute only a small number of the available signaling systems, though they mediate the environmental stress responses in fungi [5,6] and the hyphal development in pathogenic yeasts [7,8]. In plants and in the amoeba *Dictyostelium*, TCS are also responsible for several metabolic processes, such as osmoregulation, growth and differentiation [9,10]. To date, animals and humans seem to constitute an exception, as TCS have not yet been identified [11,12,13]. 

## 2. The Topology of the Two-Component Systems

TCS are important mediators of signal transduction characterized by a highly modular design, which has been adapted and integrated into a wide variety of cellular signaling events. These systems function as a basic stimulus-response coupling mechanism allowing organisms to sense and respond to distinct environmental changes and to perform a variety of functions, such as the alteration on the direction of the flagellar rotation or the regulation of the expression of a particular gene [14]. 

**Figure 1:**
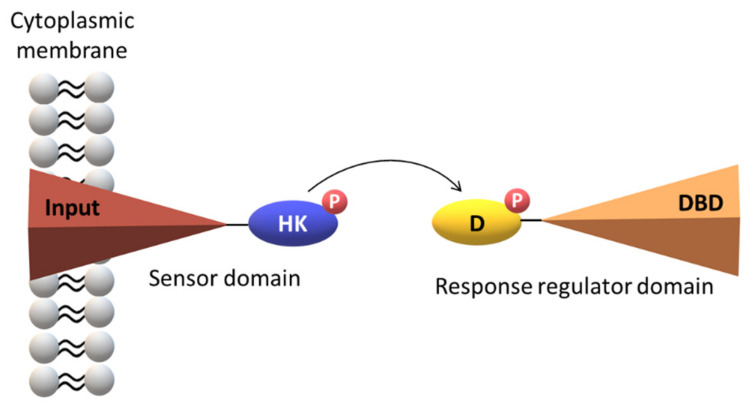
Simplified scheme for a two-component system. The histidine kinase (HK), the receiver—containing a specific aspartate residue (D)—and the DNA-binding (DBD) domains are represented.

The basic modus operandi encompasses phosphorylation events between a histidine kinase (HK) located at the sensor domain (SD) and the corresponding response regulator domain (RRD). SD is involved in the recognition of the chemical signal and the RRD triggers a particular cellular response. Both SD and RRD are composed by several subdomains (Figure 1).

**Figure 2:**
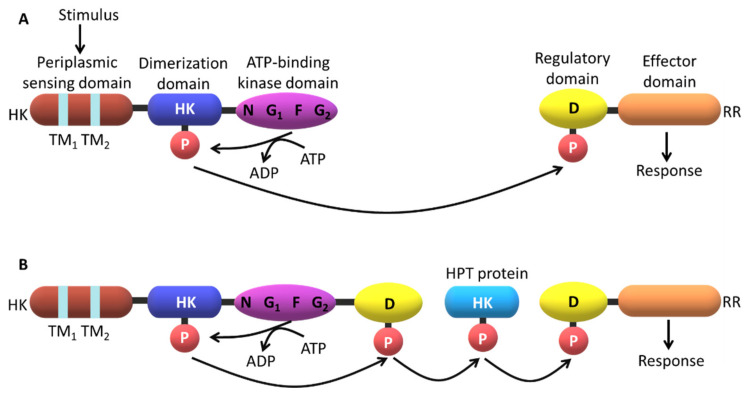
Phosphotransfer mechanisms in TCS. In both cases, the sensor domain detects the stimuli and regulates the histidine kinase domain (HK) activity. (**A**) Prototypical two-component pathway. The pathway consists of a dimeric transmembrane sensor HK and a cytoplasmic response regulator (RR) protein. A monomer of a representative HK is presented with transmembrane (TM) segments represented by TM_1_ and TM_2_. N, G_1_, F and G_2_ are conserved sequence motifs in the ATP-binding domain. HK catalyzes an ATP-dependent autophosphorylation of a specific conserved His residue within the HK dimerization domain. The phosphoryl group (P) is then transferred to a specific aspartate residue (D) at the conserved RR domain. Phosphorylation of this domain usually triggers an associated (or downstream) effector domain, which ultimately produces a specific cellular response. (**B**) A multi-component phosphorelay system often involves a hybrid HK with an additional C-terminal RR domain. In these complex systems, at least two His–Asp phosphoryl transfer events occurs, typically involving a His-containing phosphotransfer protein (HPT) operating as an His-phosphorylated intermediate.

A typical HK sensor is constituted by two domains: a N-terminal input domain and a conserved C-terminal domain that interacts with the RRD. The N-terminal domain of the input domain is considerably variable. On the other hand, the RRD contains one conserved N-terminal receiver domain and one variable C-terminal output or effector domain [13,15,16,17]. When stimulated, the sensor protein catalyzes the autophosphorylation of a conserved histidine residue via the consumption of adenosine triphosphate (ATP). Then, the HK transfers the high-energy phosphoryl group to a conserved aspartate residue at the RRD, which modulates its ability to interact with DNA sequences and trigger the appropriate cellular response (Figure 2).

In most prokaryotic systems, the response is directly conducted by the RRD that functions as a transcription factor. However, in eukaryotic systems the TCS are placed at the start of the pathways and establish an interface with more conventional signaling strategies such as mitogen-activated protein (MAP) kinase and cyclic nucleotide cascades [14]. Like most signaling pathways, the complex architecture of the TCS does not permits a direct link between the signal and the cellular response (Figure 2). 

Parallel sensory kinases affect the domain with phosphotransferase activity within a more complex phosphorelay, such as the one observed for *Vibrio cholerae* quorum sensing [18] or in the sporulation regulation in *Bacillus subtilis* (*B. subtilis*) [19]. Many eukaryotic signaling cascades involve protein kinases that undergo autophosphorylation or phosphorylate other proteins at specific serine, threonine or tyrosine residues. Examples of response regulators containing the GGDEF (guanylate cyclase protein) domain have also been identified. This includes the PleD from *Caulobacter crescentus* (*C. crescentus*) [20] responsible for the cyclic di-GMP synthesis, which is necessary for the regulation of differentiation events and biofilm formation. The efforts made to characterize signaling systems such as bacterial chemotaxis [21], aerobic/anaerobic regulation in *E. coli* [22,23], the sporulation system of *B. subtilis* [24,25] and the differentiation processes in *C. crescentus* [26,27] and *Myxococcus xanthus* [28,29] have elucidated their functions and strongly contributed to the understanding of how extracellular signals are transduced to trigger adequate cellular responses.

### 2.1. Diversity in the Sensor Domains

**Figure 3:**
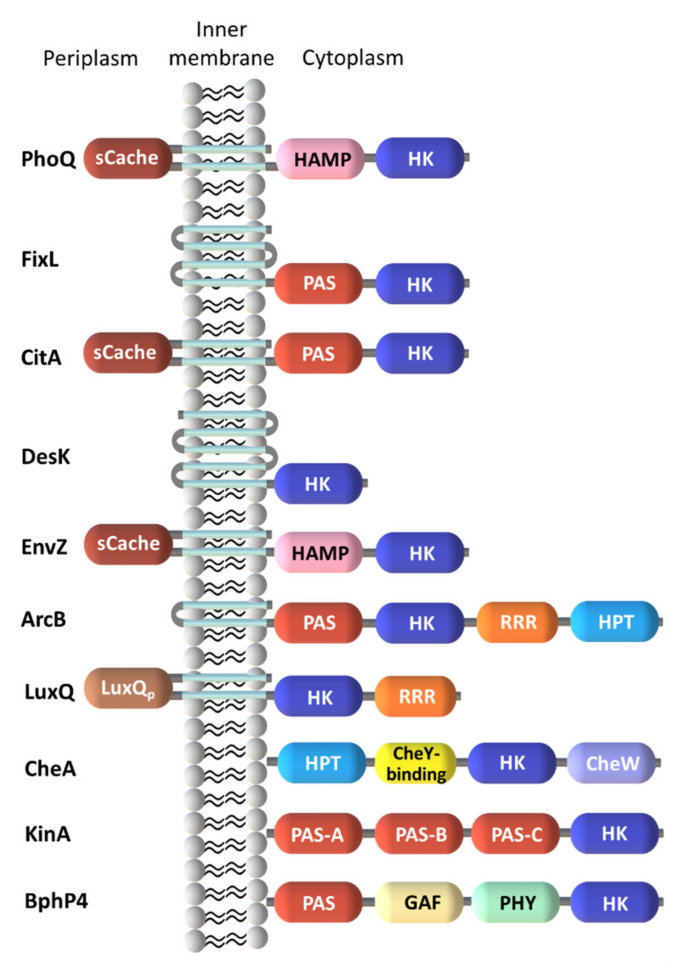
Sensor kinase domain architecture obtained from the available structural information combined with the SMART annotation (adapted from [30]). Abbreviations: sCache—single calcium channels and chemotaxis receptor; PAS—period clock protein, aryl hydrocarbon receptor and single-minded protein; HAMP—domain found in histidine kinases, adenylyl cyclases, methyl-binding proteins and phosphatases; RRR—response regulator receiver domain; HPT—histidine containing phosphotransfer; GAF domain—non-catalytic cGMP phosphodiesterase/adenylyl cyclase/FhlA-binding domain; PHY—phytochrome.

The kinase domains are part of a diverse range of SD, which allow the organisms to sense a wide range of signals, encompassing small molecules, light, pH, osmotic changes, nutrient levels, quorum-sensing proteins, redox potential and electrochemical gradients. SD are highly variable in sequence, particularly those flanked by cytoplasmic transmembrane (TM) domains [4]. In this case, stimuli are directly or indirectly sensed by these domains followed by interactions with signal transduction proteins. The precise mechanism of how the individual domains of a TCS modulates the signal response to ligand binding has not been fully elucidated even for less complex systems. However, it is well known that the variety of the SD allows multiple signals to elicit an adequate cellular response. Thus, most likely, the diversity of signals detected by the SD explains their evolution into many distinct folds (Figure 3).

Different membrane topologies were observed for SD and are divided into three main groups. The first one encompasses the cytoplasmic SD. The second group includes SD with multiple membrane-spanning helices but without an apparent extracellular domain. The stimuli detected by these HK are most likely associated to alterations of the membrane components and ion gradients. The last and largest group of SD is represented by the classical HK with extracytoplasmic domains placed between two TM helices. These SD include several families that transduce the extracellular stimulus across the membrane to regulate the activity of kinases/phosphatases. In these cases, multiple topologies can be combined for a concerted response to environmental changes.

#### 2.1.1. Cytoplasmic Sensor Domains

Multiple cytoplasmic SD have a so-called PAS domain (period clock protein, aryl hydrocarbon receptor and single-minded protein) and/or a GAF domain (non-catalytic cGMP phosphodiesterase/adenylyl cyclase/FhlA). 

**Figure 4:**
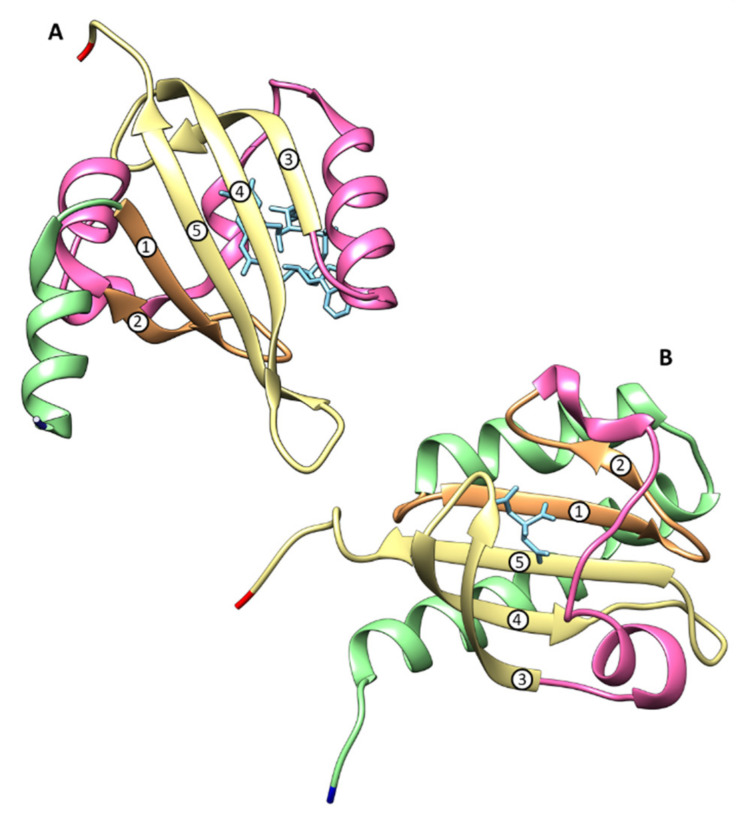
Representative structures of PAS (**A**) and sCache (**B**) domains. The first corresponds to the redox-sensing PAS domains of *Azotobacter vinelandii* NifL (PDB ID: 2GJ3) bound to FAD (flavin adenine dinucleotide) and the second to the ligand-binding domain of the *Klebsiella pneumoniae* (*K. pneumoniae*) sensor kinase CitA protein (PDB: 1P0Z). The core of β-strands are labeled with Arabic numbers. The structures were generated with CHIMERA [38] and are colored as follows: N-terminal end—dark blue; the first α-helix region—green; the β-strands 1 and 2—orange; the inter-domain α-helix region—pink; the β-strands 3 to 5—yellow and the C-terminal—red. FAD and citrate molecules are shown in light blue.

These domains typically contain between 100-120 amino acids [31,32,33] and are structurally characterized by a five stranded β-sheet combined with α-helices [31,34]. Cytoplasmic PAS domains show a α-2β-4α-3β topology (Figure 4A) whereas extracytoplasmic domains, previously named PAS-like, PDC (PhoQ, DcuS and CitA) and more recently Cache (calcium channels and chemotaxis receptor [35]) show a 3α-2β-1/2α-3β-α topology **(**Figure 4B) [34,36,37].

A different structural family motif found within cytoplasmic SD corresponds to the GAF fold [39]. This consists of a six stranded anti-parallel β-sheet core, which is structurally related to PAS domains and is typically present in the HK sensors. Both domains are highly variable in their amino acid sequence and structure, a feature that confers high versatility to the domains responsible for the stimuli recognition and signal transduction.

Phytochromes from fungi, bacteria and plants constitute the third family of cytoplasmic SD. This family includes soluble HK photoreceptors. Structural data were obtained for the *Pseudomonas aeruginosa* (*P. aeruginosa*) bacteriophytochrome photosensory core domain (PaBphP-PCD) and revealed a tripartite photosensory core domain (PCD) containing PAS, GAF and phytochrome (PHY) domains [40,41]. Like the PAS and GAF domains, the PHY domain, in addition to helical elements, also contains a five-stranded anti-parallel β-sheet scaffold. On the other hand, the PAS and GAF domains possess a chromophore-binding domain (CBD) that covalently binds a bilin chromophore responsible for the photoconvertion. Structural data were reported for the CBD from *Dienococcus radiodurans* DrBphP-CBD [42], the RpBphP-CBD from *Rhodopseudomonas palustris* [43] and PCD from *Synechocystis* 6803 Cph1 [44].

#### 2.1.2. Membrane-Embedded Sensor Domains

HK receptors containing several TM segments frequently miss an obvious extracellular domain. In these cases, the sensing region is found within the TM helices. Complementary biochemical and biophysical studies have confirmed this hypothesis in the case of the DesK temperature sensor [45,46]. In the case of the membrane-embedded SenS redox sensor, it was also shown that binding of an octameric heme-binding protein to the N-terminal TM region is crucial to trigger a cellular response [47]. More examples are provided by the plant Etr1 HK [48] and AgrC quorum sensor [49,50], which respond to the interaction between ethylene and an autoinducing peptide in the TM regions, respectively. Structures for TM regions of HK receptors are still scarce. However, some highlights of this type of arrangements were provided by the structural data obtained for the phototaxis sensory rhodopsin II-transducer complex (HtrII-SrII) [51].

#### 2.1.3. Extracytoplasmic Sensor Domains 

Three classes of extracellular SD have been established according to their sequence similarity and fold [17,52]: (i) α-helical fold like the four-helix bundle (4HB) NarX and Tar detector domains from *E. coli*, (ii) β-sheet fold like the *P. aeruginosa* RetS periplasmic detector domain and (iii) a mixed α/β fold named PAS-like/PDC [53] and, more recently as Cache [35]. The amino acid sequences are highly variable in extracytoplasmic SD. Thus, only few were recognized as periplasmic: the periplasmic solute-binding protein (PBP), the Cache domains, the cyclases/histidine kinases associated sensory extracellular (CHASE) domains and nitrate/nitrite sensor (NIT) domains [54,55].

**Figure 5:**
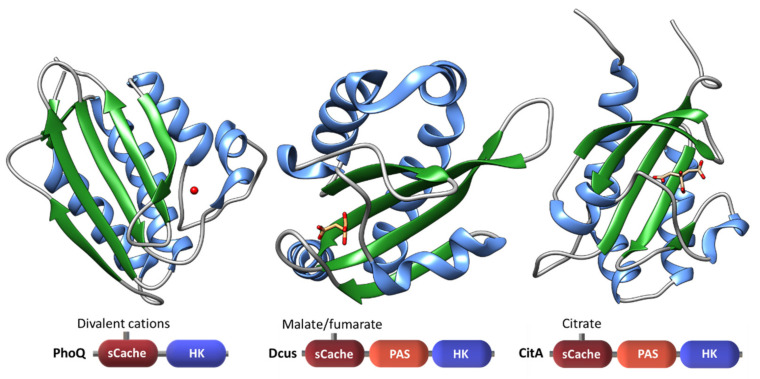
Structure of the sCache domains of PhoQ (PDB: 1ID0) from *Salmonella enterica*, DcuS (PDB: 3BY8) from *E. coli* and CitA (PDB: 1P0Z) from *K. pneumoniae*. The ligands of each sCache domain are also represented: Mg^2+^ (red), malate (coloured by element) and citrate (coloured by element). The β-strands and α-helices are colored green and blue, respectively. The sensor domain architectures are shown at the bottom of figure.

The new nomenclature proposed for extracytoplasmatic PAS-like or PDC domains—Cache—is based on an extensive bioinformatic study carried out by Upadhyay and co-workers [35] in which the available structures of several bacterial SD were compared. The authors found that the Cache domain is homologous to, but distinct from the PAS superfamily. Thus, Cache sensors define a larger discrete domain and their sequence diversity suggests an independent parallel evolution from PAS domains [17,35]. The Cache domains exist either in a mono-modular (sCache) or bi-modular (dCache) configuration and are the most abundant sensor domains in histidine kinases and chemoreceptors. Structures of Cache domains are typified by the CitA, DcuS and PhoQ sensors (Figure 5).

Additions to the structural collection of sCache sensors are provided by the oligosaccharide sensor AbfS [56] and the PhoR periplasmic domain [36]. 

The activities of some sensors are regulated by auxiliary proteins. In these cases, the binding of such proteins is in fact the primary ligand-binding event. The structures of some proteins that are responsible for the control of their respective sensor kinases have been determined: LuxP that regulates the LuxQ activity; YycH and YycI from *B. subtilis* that regulate the YycG sensor kinase [57,58] and the cytoplasmic proteins pXO1-118 and pXO2–61 from *Bacillus anthracis* that control the sporulation [59].

#### 2.1.4. Heme-Based Sensors

Heme proteins are one of the most versatile group of proteins in Nature. In addition to their well-known role in electron transfer, oxygen transport or reduction of peroxides, they can also be involved in chemotaxis and regulation of gene expression [60]. The heme iron, at the porphyrin ring, can present different redox states, most commonly Fe^2+^ and Fe^3+^. The iron atom can be penta- or hexacoordinated and, depending upon the axial ligands, it can be high-spin (HS) or low-spin (LS). The interconversion between different spin states, as well as between different oxidation states can be explored by the SD to trigger an adequate cellular response. 

The currently known heme-based sensors are grouped into six different families according to their heme-binding domains: (i) heme-nitric oxide binding domain (H-NOX), (ii) globin-coupled sensor fold, (iii) heme binding PAS fold, (iv) GAF domain (v) bacterial CO oxidation transcriptional activator (CooA) homologues, and (vi) the sensor containing heme instead of cobalamin (SCHIC) domain of anoxygenic phototrophic proteobacteria [61]. All these proteins contain a heme group, but the architecture of their domains is structurally diverse. The most representative structural motif of this group of sensors is the PAS fold [62,63,64]. 

The structural variability of the heme-containing sensors correlates with their diverse role in living systems. Heme groups have been reported in PAS domains (FixL in *Rhizobium meliloti* and EcDos in *E. coli*), GAF domains (*Mycobacterium tuberculosis* (*M.* *tuberculosis*), DosS and DosT) and in globin domains (*B. subtilis* HemAT). The PAS domains usually contain heme cofactors, while GAF domains frequently have cyclic nucleotides.

Considering the remarkable biological relevance of small diatomic molecules, as well as the efficient adjustments of most organisms to environmental fluctuations, it is expected that the common functions of heme-based sensors are well adapted to the sensing of such molecules, including CO, NO or O_2_. The discrimination between diatomic ligands with similar electronic properties is critical to trigger an adequate cellular response [65].

##### *b*-Type Heme Sensor Domains

**Figure 6:**
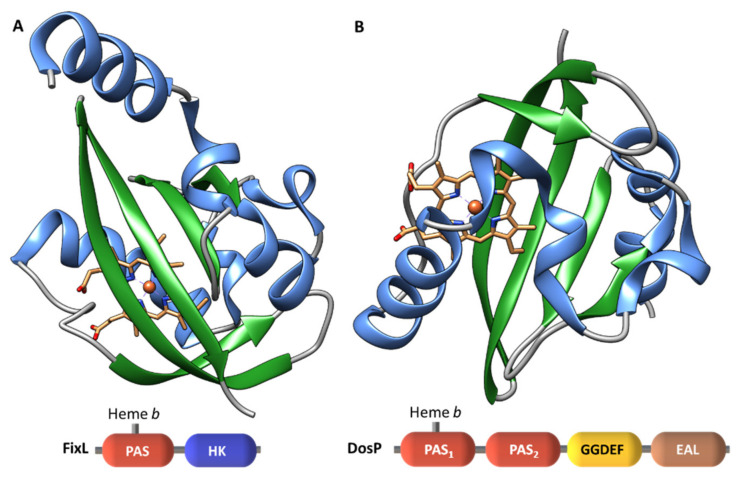
Structure of FixL (**A**) and DosP (**B**) PAS sensor domains. β-strands and α-helices are represented in green and blue, respectively. The hemes are colored by element.

FixL is a heme-based oxygen sensor which controls the expression of specific proteins under microaerobic or anaerobic conditions [66,67,68] and contains one *b*-type heme group [69]. The same type of heme is found in the oxygen sensor DosP (EcDOS) from *E. coli* [70]. This sensor functions as an oxygen-regulated phosphodiesterase and catalyzes the cleavage of cyclic di-GMP into the linear dinucleotide pGpG [71,72]. A conserved histidine residue is responsible for the heme binding in FixL-PAS and DosP-PAS_1_ (Figure 6), suggesting a similar evolutionary origin for these oxygen sensors. Other examples of SD containing *b*-type heme groups are the GAF domain (GAF-A) of *M. tuberculosis* DosS [73] and DosT [74], which detect modifications in the redox state of the iron caused by the binding of oxygen. 

##### *c*-Type Heme Sensor Domains

In contrast with *b*-type hemes, few TCS containing *c*-type hemes have been identified to date. One example is the multidomain protein MA4561 from *Methanosarcina acetivorans* [75], which is predicted to be a PHY-like photoreceptor with a HK-like output domain. More recently the MA4561 was identified as the sensor kinase of a TCS regulation of methyl sulfide methyltransferase system and designated as methyl sulfide methyltransferase-associated sensor (MsmS). 

The *Thermosynechococcus elongatus* protein Tll0287 has a PAS-like domain with a *c*-type heme and was identified as a TCS involved in signal transduction in response to environmental factors related to the sulfide metabolism [76].

PAS domains containing *c*-type hemes were identified in chemoreceptor proteins from *Desulfovibrio vulgaris* (*D. vulgaris*) Hildenborough (DcrA) [77] and *G. sulfurreducens* (GSU0303, GSU0582 and GSU0935) [78,79]. DcrA chemotaxis signal transducer protein contains a periplasmic domain with a *c*-type (DcrAN) responsible for sensing redox potential and oxygen variations. It is the first example of a heme-based sensor protein containing a *c*-type heme reported in the literature. This protein has a cytoplasmic PAS domain without the heme cofactor. Thus, it was proposed that the cytoplasmic PAS domain of DcrA may be involved in the output response to the signal sensed by the heme *c* in the periplasmic space.

### 2.2. Diversity in the Response Regulator Domains

In prokaryotes, RRD are usually the last components of the signaling pathways and are directly involved in the effecting responses. On the other hand, in eukaryotes, RRD act as intermediates and establish the necessary interface with proteins that regulate kinase cascades or the production of nucleotide-based second messengers [80]. Only a small number of prokaryotic RRD are conserved. The RRD are highly diverse and can be divided into several families, such as those containing DNA-binding effectors, RNA-binding domains or enzymatic domains. Structural data have been obtained for the different sensors showing that different subfamilies can be defined. In the case of DNA-binding effectors, different structural motifs are provided by the OmpR/PhoB [81], the NarL/FixJ [82], the NtrC/DctD AAA + ATPase domain [83] and the LytTR domain [84]. RNA binding domains are mainly from the ANTAR (AmiR and NasR transcription antitermination regulator) family that function as anti-termination factors [85]. Most of the enzymatic domains regulate the cyclic diguanylate (di-GMP) synthesis or hydrolysis. The remaining enzymatic subfamilies are chemotaxis methylesterase CheB domains that are frequently linked with other PAS or GAF domains. Many additional enzymatic domains in RRD are less frequent. A minor and heterogeneous group that mediates protein–protein or protein–ligand interactions uncommon. Structural data have been obtained for some members of this group of sensors [86,87,88,89,90,91]. This restricted group is dominated by the chemotaxis CheV-like proteins. Hnr-like regulators of stress sigma factor RpoS (RNA polymerase sigma S), HPT (histidine containing phosphotransfer), PAS, GAF and cyclic nucleotide-monophosphate (cNMP) binding domains are examples of other subfamilies. 

### 2.3. Signaling Mechanisms

**Figure 7:**
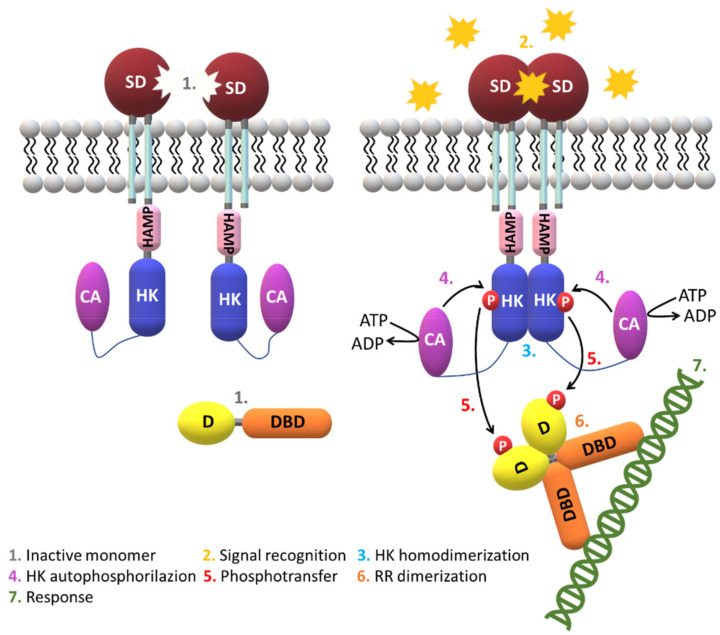
Prototypical TCS signaling pathway. A variety of extracellular signals are detected by the SD, which triggers the membrane-bound HK dimerization. The TM helices form four-helix bundles in the HK dimeric states. The signaling mechanism then involves the autophosphorylation of HK mediated by hydrolysis of ATP and concomitant phosphorylation of a conserved histidine by the catalytic (CA) domain. This then leads to the phosphotransfer of the HK phosphoryl group to a cytoplasmatic response regulator (RR) that is composed by two domains: the receiver domain (D)—that recognizes and binds to the HK domain—and the effector DNA-binding domain (DBD) that modulates the expression of target genes and hence the cellular response. Upon phosphorylation, the RR is activated and undergoes conformational changes that promote dimerization or more ordered oligomerization states that favor the interaction between the RR and bacterial DNA. Alternatively, the RR may also act as an enzyme, such as a methylesterase or an ATPase. TCS signaling is terminated by dephosphorylation of the RR, which can be auto induced or mediated by the HK or by auxiliary proteins. The CA and the dimerization domains are conserved and found in all HK, whereas the remaining signaling domains (HAMP, GAF, PAS and PHY) are variable.

It has been suggested that signal transduction via HK domains mostly requires the formation of a dimer. However, several SD transmit the signal in their monomeric form (NarX, DcuS, DctB, CitA) [53,92,93,94]. The monomer self-association has been measured in some cases (TorS and DcuS) [95,96] and is a process that requires high protein concentration. Using light scattering experiments in the LuxPQ sensor complex it was also possible to conclude that the level of dimerization in solution depends on the signaling state and that variations in affinity at the dimers’ interface is important [97]. The experiments suggested that signaling is not mediated by the dimerization of HK domains. Instead, the detection of the stimuli may alter the extent of the protein–protein interactions with the surface of the pre-formed HK dimer, stabilize this conformation and transmit the structural changes along the dimer interface to the core domains (Figure 7). 

Structural data obtained in recent years for several extracytoplasmic and cytoplasmic SD permitted to establish different classes of proteins sharing similar folds. 

The structural information obtained for Tar and systems like EnvZ and NarX, suggested the existence of a piston-like movement that propagates the signaling via the TM helices into the cytoplasm [98,99]. However, the exact conformational changes that occur in this process are still unknown. Another example was provided by the studies carried out in the quorum sensing LuxQ from *Vibrio harveyi*. In this case, the ligand binds to a periplasmic protein (LuxP), which then drives the formation of an asymmetrical dimer that transmits the signal to cytoplasmic domains and inhibits the kinase activity [97]. In contrast with this indirect mechanism, direct mechanisms have been identified for a series of proteins—for example, PhoQ proteins from *E. coli* and *Salmonella enterica* or CitA from *K. pneumoniae*.

The physiological relevance of the proteins’ quaternary structure remains puzzling since isolated Cache domains have smaller propensity for dimerization and their plasticity creates distinct orientations of the dimers’ subunits in the crystals. On the other hand, such heterogeneity might reveal distinct signaling stages and hence contain valuable information.

Some Cache structures suggest a common feature of the N- and C-terminal regions for these periplasmic SD by placing the TM helices at locations consistent with a four-helix bundle matching those of Tar and NarX [92,100,101]. Curiously, citrate binding to CitA triggers the sensory domain contraction, coherent with a TM helical piston-like motion. On the other hand, the structural data available for HAMP domains, dominated by helical coiled coils suggests a helical mechanism [40,44,74,102,103,104,105,106,107]. However, in any case, it is still unknown how the movements of TM helices are converted to helix rotation. Specific conformational modifications are indeed caused by binding of ligands and cofactors, but how these variations trigger a precise enzymatic activity is still, in most cases, remains to be clarified.

## 3. A Specialized Two-Component System for Chemotaxis

Chemotaxis is intimately linked to bacterial growth and survival and is the key for the movement towards environments containing either higher concentrations of beneficial compounds or lower concentrations of toxic chemicals. The bacterial chemotaxis machinery was firstly identified in *E. coli* and *Salmonella enterica serovar* Typhimurium for the regulation of their flagellar-based motility [108,109,110]. Most sequenced bacterial genomes include homologous genes encoding for flagella and chemosensory pathways’ components. This suggests that motility is widespread and provides environmental advantages, particularly in non-homogeneous and nutrient-limiting habitats. However, there are reports showing that the two type of genes are not always simultaneously present in the same microorganism [111]. This suggests that the ability to move is not, by itself, essential and must be predisposed by the proliferation of certain bacteria in specific environments. 

Nearly 50 years ago, Julius Adler and Margaret Dahl [110] observed that the *E. coli* B275 strain required L-methionine to grow and perform chemotaxis. Using radioactive methionine, the authors showed that the methyl group is incorporated into a protein located in the cytoplasmic membrane—the methyl-accepting chemotaxis protein [112]. As a result, methyl-accepting chemotaxis proteins (MCP) or transducer-like proteins (TLP) were identified as the bacterial chemotaxis receptors. The first identified sensory chemoreceptor was designated as MCP I (or Tsr serine receptor). This was followed by the identification of a cytoplasmic membrane protein—the MCP-like receptor Aer for oxygen taxis (aerotaxis) [113,114,115,116].

MCP are the predominant chemoreceptors in bacteria and have been found in many *Archaea*. The best investigated is the Tar chemoreceptor from *E. coli*, which has four MCP (Tar, Tsr, Tap and Trg) and one MCP-like protein (Aer). Roger Alexander and Igor Zhulin [117] identified 2125 MCP sequences in 152 genomes of *Bacteria* and *Archaea*. Barbara A. Methé and co-workers [118] discovered multiple homologs of chemotaxis genes (~70) in *G. sulfurreducens* genome, 34 MCP genes and six major *che* gene clusters. 

**Figure 8:**
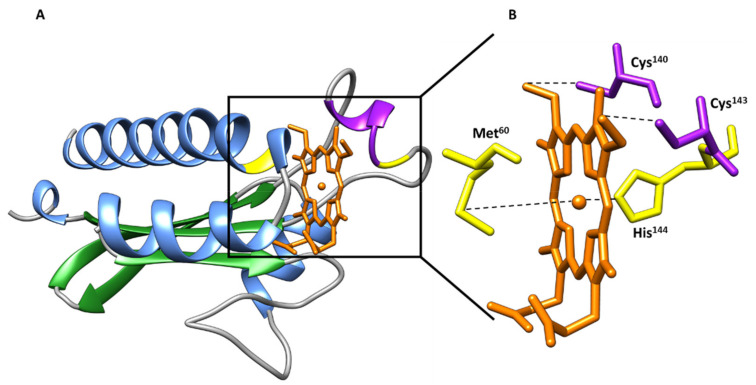
(**A**) Structure of the heme pocket region of MCP GSU0935 sensor domain from *G. sulfurreducens* and (**B**) schematic representation of heme group (in orange) with its binding residues (axial ligands—Met^60^ and His^144^—and cysteine residues from the heme binding motif—Cys^140^ and Cys^143^). The β-strands and α-helices are represented in green and blue, respectively. The heme-binding site is represented in purple and the axial ligands in yellow.

Until 2003, the only structure of a periplasmic ligand-binding domain available was the one of the chemoreceptor Tar that encompasses a four-helix bundle motif [119]. From the MCP predicted by *Geobacter metallireducens* (*G. metallireducens*), *G. sulfurreducens* and *Geobacter uraniireducens* (*G. uraniireducens*) genomes, 90% have two TM helices. The majority (80%) contain periplasmic domains with about 150-200 amino acids and are comparable to the periplasmic domains of *E. coli* MCP Tsr [120]. While the architectures of most of these MCP are unknown, those of two MCP from *G. sulfurreducens* (GSU0935 and GSU0582) were established and revealed PAS-like domains containing *c*-type heme groups [78] (Figure 8).

**Figure 9:**
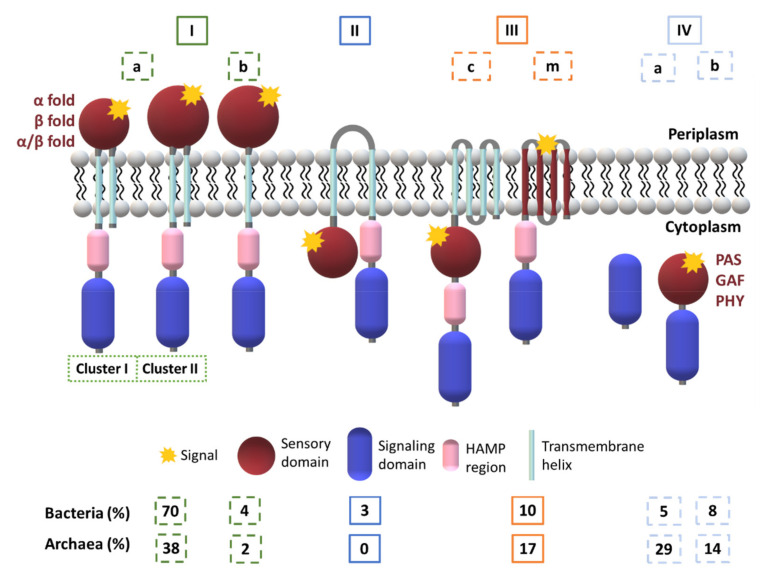
Classification of MCP according to Lacal and co-workers [52].

The membrane topology appears to be an important feature for the MCP’s function and hence four major classes were defined (I–IV) [121] (Figure 9). 

Later, Wuichet and co-workers [122] have subdivided the class III into class IIIc and IIIm, according to the nature of the ligand binding domains. Subsequently, Lacal and co-workers [52] expanded the classification to seven different topologies (Ia, Ib, II, IIIm, IIIc, IVa and IVb). The class I was subdivided based on the number of TM helices and class IV according to the presence or absence of ligand binding domains. Depending upon the number of amino acid residues of the SD, subclass Ia has been further divided into clusters I and II [52]. 

The members of class I have a periplasmic ligand-binding domain placed between two TM helices and a cytoplasmic component containing the methylation sites and a conserved domain that regulates the HK activity [121,123,124]. This is a predominant class of MCP in Bacteria and Archaea [121]. 

Class II receptors harbor a single hydrophobic patch between a putative SD, a cytoplasmic HAMP region and a cytoplasmic signaling module. The class II receptors (e.g., Aer redox transducer) are found solely in Bacteria and are much less abundant (3%) compared to the members of the other classes [52]. 

Transducers from class III have a distinct number of membrane-spanning helices with the SD placed either within the membrane (subclass IIIm) or in the cytoplasm (subclass IIIc). In both cases, the SD is followed by the cytoplasmic HAMP region and the signaling domain. The subclass IIIm lacks an extracellular domain and the stimuli is most likely membrane-associated. The photoreceptor from *Synechocystis* is a typical representative of class III [125]. The patch may consist of a single hydrophobic region or contain several TM regions.

The MCP from class IV are cytoplasmic containing (subclass IVa) or not (subclass IVb) an identifiable SD. These MCP lack TM helices as well as, in most cases, the HAMP domains. The mechanism of ligand recognition in subclass IVb is still under debate.

There are some hybrid systems identified for classes II and III, which combine periplasmic and cytoplasmic SD [126]. 

MCP sequence comparison indicates that the SD tend to be highly variable, whereas the cytoplasmic signaling domains that interact with the downstream regulatory proteins are highly conserved. The middle components of the chemotaxis signaling pathway, placed between the MCP receptors and the flagella are the Che proteins (Table 1). 

The *che* genes are widespread in Bacteria and Archaea. The genes respond to the MCP’s interaction with an attractant or repellent. CheA, CheW, CheY and CheZ transmit the flagellar response. Subsequently, CheB and CheR conduct the adaptation to the stimulus by methylating or demethylating the MCP. Receptor MCP are coupled to an adaptor protein CheW (or CheV), to a CheA HK, and two RRD (CheB and CheY) that compete for binding to CheA. CheY is a specialized single-domain flagellar motor-binding protein that regulates the response, whereas CheB has two domains, one responsible with methylesterase activity and controls the adaptation of the MCP and a second one that functions as a response regulator [127,128]. The MCP contacts with CheW and CheA and regulates the phosphorylation of the kinase [129,130]. Then, CheA∼P phosphorylates CheY (CheY~P) which subsequently interacts with its target—the flagellar motor—to alter the direction of the motor rotation [131,132]. CheA∼P also phosphorylates the CheB (CheB∼P) that demethylates the highly conserved helical segments (Glx-Glx-X-X-Ala-Ser/Thr, where Glx is either a glutamine or a glutamate residue), induces additional conformational changes and adapts to the receptor. In *E. coli* chemotaxis, an auxiliary protein (the phosphatase CheZ) oligomerizes with the CheY∼P and increases the spontaneous dephosphorylation rate of CheY∼P for rapid signal termination [133].

**Figure 10:**
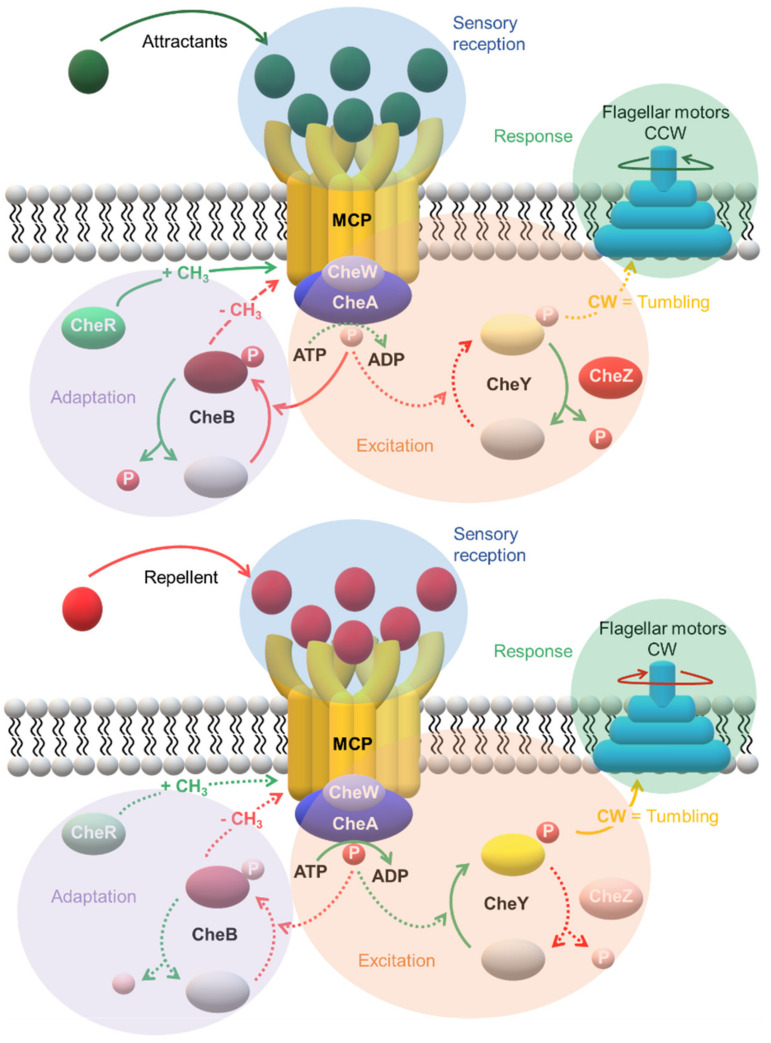
Mechanism of chemotaxis in *E. coli*. The chemoreceptors function as trimers of dimers. The rotation of the flagellar motor is regulated by the ratio of the phosphorylated form of CheY (CheY~P) versus its dephosphorylated form (CheY). Steady dephosphorylation of CheY~P by the phosphatase CheZ maintains a constant ratio of CheY~P/CheY and a basal level of alternating counterclockwise (CCW, causing cells to swim more straight runs—moving upwards the attractant gradient) and clockwise (CW, causing cells to tumble) rotation. This equilibrium is affected either by attractants or repellents. In the first case, the phosphorylation of CheA is blocked and the high levels of CheY favor the CCW rotation (see dashed arrows in the upper panel). On the other hand, repellents decrease the CheZ activity and increase both the levels of CheA~P and CheY~P. As a consequence, CW rotation is favored. Both processes are called excitation. A methylation feedback loop on the MCP re-establishes the basal CheY~P/CheY ratio (adaptation).

The cells’ capability to detect and respond to small concentration changes of attractant and repellents is intimately associated to the diversity of their chemoreceptors. The levels of CheY∼P increase with the decrease of the attractant. Consequently, the flagellar motor switches to clockwise rotation and alter the cell tumbling and direction. On the other hand, the demethylated MCP, as a response to the higher levels of CheB∼P, reduces the autophosphorylation rate of CheA and therefore the switch rate of the flagellar motor rotation reverts to the pre-stimulus state. The system is then adapted and ready to sense any subsequent variation on the signal molecule(s). On the other hand, an increase in the concentration of the attractant will inhibit the autophosphorylation of CheA and decrease the frequency of the motor switching, in response to lower levels of CheY∼P. This event triggers the bacterium to swim in this new direction. Then, CheB∼P decreases, which favors the methylation of MCP by the methyltransferase CheR. This brings about the CheA autophosphorylation to the pre-stimulus state and allows the bacterium to restore its typical direction changing (Figure 10).

## 4. The Multiple Chemosensory Systems in *Geobacter* Bacteria

*Geobacter* are facultative anaerobes Gram-negative δ-Proteobacteria with extraordinary respiratory diversity. *Geobacter* are abundant in soils and sedimentary habitats capable of completely oxidizing organic compounds to CO_2_ [134]. Many of the electron acceptors used by *Geobacter* are insoluble under environmental conditions. Due to their ability to transfer extracellular electrons, these bacteria are considered as attractive targets for several practical applications for bioremediation of radioactive/toxic metals in contaminated subsurface environments and for bioenergy generation by converting organic compounds into electricity in microbial fuel cells [135,136,137]. Given the variety of electron acceptors and the diverse habitats in which *Geobacter* cells proliferate these organisms contain a high number of MCP chemoreceptors. The ability to respond efficiently in extreme environments, such as the saturation of a specific electron acceptor, the replacement by different electron donors or even changes in the surroundings redox potential, is an essential feature of *Geobacter* bacteria, which promote well-organized metabolic adjustments.

Recent analysis of sequenced *Geobacter* genomes revealed an abundance of *che* gene homologs and multiple chemotaxis systems [120] (Table 2). 

*G. sulfurreducens* PCA was the first *Geobacter sp*. for which the genome was fully sequenced [118] and for which a genetic system was developed [139]. The analysis of the genome sequence revealed twelve proteins identified as signal transducers with one (GSU0283, GSU0303, GSU0356, GSU0582, GSU0935, GSU1302, GSU2314, GSU2622, GSU2816, GSU2916) or two *c*-type heme-binding motifs (GSU0591, GSU0599), with an amino acid signature of CX_2–4_CH. Eight of these proteins are annotated as two-component system proteins (GSU0283, GSU0303, GSU0356, GSU0599, GSU1302, GSU2314, GSU2816, GSU2916), two as chemotaxis signal transducer proteins (GSU0582 and GSU0935), one as cytochrome *c* family protein (GSU0591) and one as HAMP/GAF/HD-GYP protein (GSU2622) [79,140]. Amongst these *G. sulfurreducens*’ signal transduction proteins, nine have homologs in *G. metallireducens* [141].

### 4.1. Topology of Heme MCP in G. sulfurreducens

**Figure 11:**
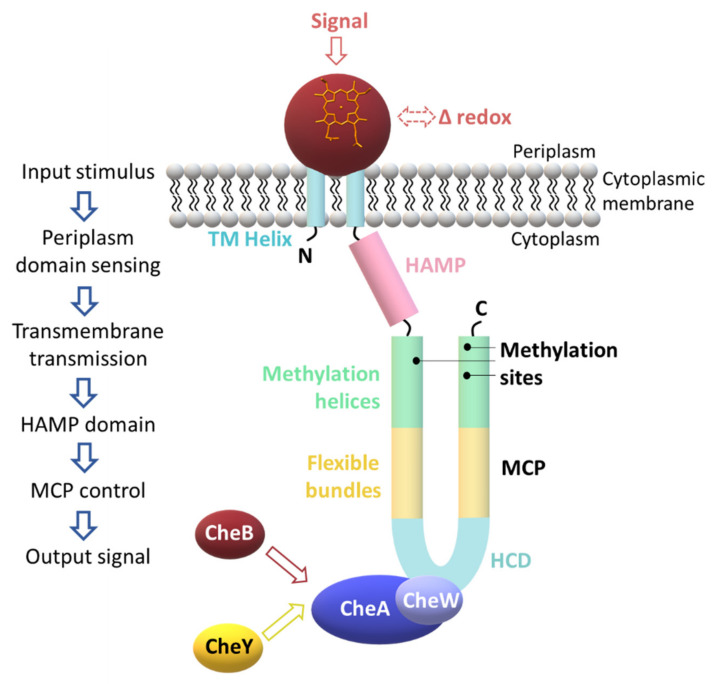
Proposed sensing mechanism of *G. sulfurreducens* heme MCP sensors. The periplasmic domain senses the external signal, which might be related with the redox sensing and transfers the signal through the TM helices, activates the HAMP domain and then the MCP. Then, the highly conserved signaling domain (HCD) is activated and regulates the ratios of CheW/CheW~P and CheA/CheA~P (see also Figure 10). The response regulators involved in chemotaxis, CheY and CheB, compete for CheA binding. CheY interacts with the flagellar motors and controls the direction of the motor rotation, while CheB demethylates specific and highly conserved segments located in MCP (methylation sites), thus controlling the adaptation for a ligand-bound receptor complex.

The sequence alignment of the MCP heme sensors GSU0582 and GSU0935 indicates that they have a periplasmic domain containing a heme *c*, connected via two TM to the cytoplasmic domain, which consists of a HAMP domain followed by a MCP domain. The proposed action mechanism involves the sensing of the external stimulus by the periplasmic SD that then transmits the signal via the TM helices, activates its cytoplasmic transducer domains and triggers the response to the original extracellular signal [78] (Figure 11).

### 4.2. Structural Features of MCP Containing Heme Groups in G. sulfurreducens

**Figure 12:**
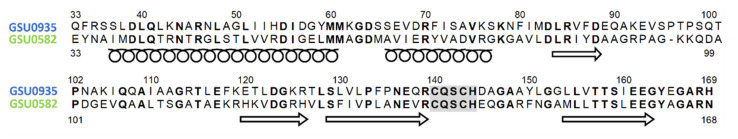
Amino acid sequence alignment of sensor domains GSU0935 and GSU0582 from *G. sulfurreducens*. The conserved residues are indicated in bold face and the heme binding motif in a gray box. The alignment of the proteins was performed with the basic local alignment search tool (BLAST) [142]. Helical and strand segments are indicated according to PHYRE automatic fold recognition server for secondary structure prediction [143].

The sequence alignment of the GSU0582 and GSU0935 heme SD is represented (Figure 12).

**Figure 13:**
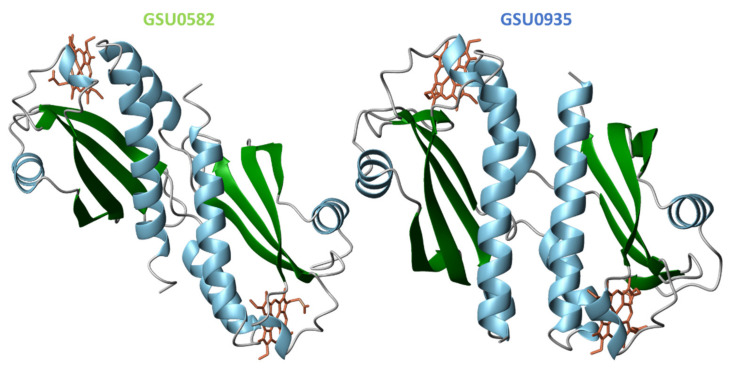
Structure of the sensor domains GSU0582 (PDB: 3B47) and GSU0935 (PDB: 3B42) in the oxidized form. α-helices and β-strands are represented in green and blue, respectively. The heme groups are represented in orange.

Despite the moderate sequence identity (40%) of the two sensor domains, the crystal structures [78] showed that both sensors form swapped dimers from two distinct protein chains with a PAS-like fold (Figure 13). The structures were obtained in the oxidized state and constituted the first example of PAS-like domains with *c*-type hemes. 

The swapped region encompasses two α-helices at the N-terminal and in the dimer the hemes are placed between the α-helices of one monomer and the β-strands of the other (Figure 13). In the structure of the GSU0582 dimer, each heme is HS and axially coordinated by His^143^ and by a water molecule. On the other hand, in GSU0935 dimer, one of the hemes is HS with axial ligands His^144^ and a water molecule while the other heme is LS—hexacoordinated with His^144^-Met^60^ ligation. Complementary spectroscopic experiments, including electron paramagnetic resonance (EPR), nuclear magnetic resonance (NMR) and optical absorption spectroscopy showed that the hemes are HS and LS in the oxidized and reduced forms, respectively. It was also shown that the interconversion between the HS and LS forms is driven by the replacement of the heme distal axial ligand (see below) [78].

**Figure 14:**
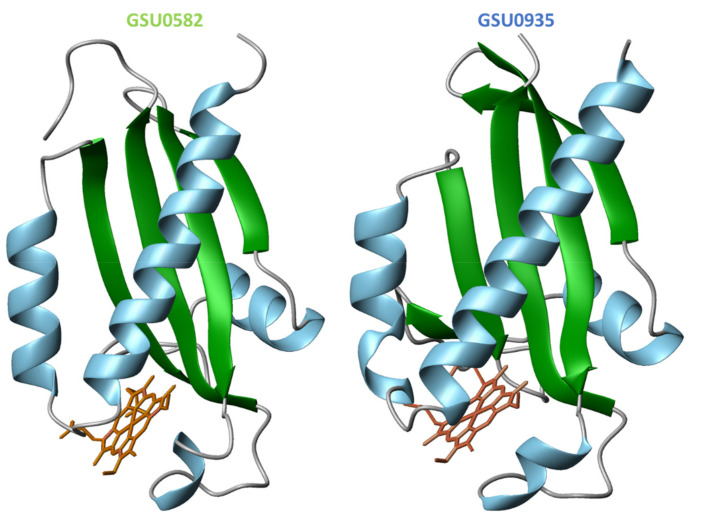
GSU0582 and GSU0935 predicted monomer models constructed using the program 3D-PSSM. The two helical segments at the N-terminal followed by four-stranded antiparallel β-sheets are represented in blue and green, respectively. The heme groups are represented in orange.

Since dimers are formed at the protein concentrations used in the crystallization studies, to date no structural information is available for the monomeric form of these sensor domains. A monomer model was predicted using the program 3D-PSSM and its fold resembles the one of CitA from *K. pneumoniae* (Figure 14) [78].

From the comparison of the monomers and swapped dimer structures (*cf.* Figure 13 and Figure 14) it is evident that the swapped dimerization mechanism brings considerable conformational changes [78].

### 4.3. Spectroscopic Features of Heme MCP in G. sulfurreducens

The functional properties of *G. sulfurreducens* GSU0582 and GSU0935 heme sensors were studied by spectroscopic techniques. Both *c*-type heme-containing sensors display similar spectroscopic features, as studied by UV–visible, resonance Raman, EPR and NMR spectroscopies [144]. These studies showed that the heme group is HS in the oxidized form (*S* = 5⁄2) and LS (*S* = 0) in the reduced form. This spin change is probably caused by the binding of Met^60^. It was also showed that both sensors can bind carbon monoxide (CO) in the reduced form but not in the oxidized form. On the other hand, nitric oxide (NO) binds in both forms. The binding of CO and NO to the reduced heme replaces the axial Met^60^ and the binding/dissociation of the two diatomic molecules is fully reversible. 

The data obtained from resonance Raman experiments, molecular dynamics calculations and binding studies revealed several common features for both sensors: (i) co-existence of two spin populations in the oxidized state; (ii) reduction of the heme favors the LS population; (iii) CO binding forms a hexacoordinated (6c) LS-CO species; (iv) NO binding forms yields a pentacoordinated (5c) HS-NO and 6cLS-NO species in both redox states. The data also suggested that in the reduced form the sensors are unable to discriminate between the two diatomic molecules, whereas in the oxidized GSU0582 has a much higher affinity for NO [144]. Overall, the detailed characterization of GSU0582 and GSU0935 suggests that the signal transduction mechanism in these sensors is based on a redox-linked heme spin state/coordination alteration. The resonance Raman data confirmed that the axial methionine binds strongly to the reduced form of the heme that is detached only in the oxidized state. Thus, most likely the sensors are inactive in the reduced form and become active when the environment redox potential leads to the oxidation of the heme iron with the concomitant release of the axial methionine [144].

**Figure 15:**
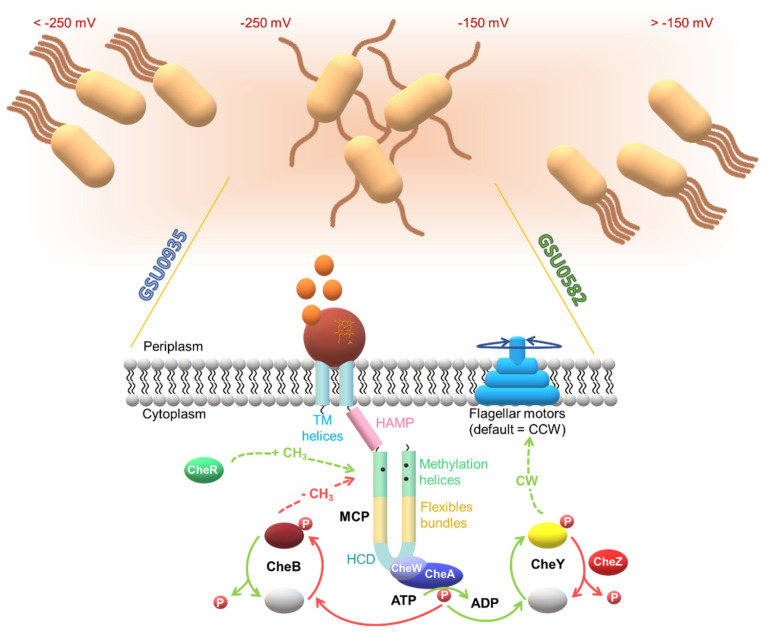
Model for the signal transduction mechanism mediated by MCP in *G. sulfurreducens*. Signal sensing promotes the phosphorylation of CheA coupled by an adaptor protein CheW. The cascade continues with the phosphorylation of CheY. CheY~P is responsible for clockwise flagellar rotation (CW) and tumbling movements dominate. On the other hand, signal saturation blocks the cascade and the reverse events occur. In this case instead of tumbling, running movements predominates because of the counterclockwise flagellar rotation (CCW). Once a directional flagellar motion is no longer necessary, the phosphatase CheZ oligomerizes with the phosphorylated CheY and increases the spontaneous dephosphorylation rate of CheY∼P. Then, adaptation follows: CheB causes demethylation of the methylated MCP, while CheR causes its methylation.

Despite the structural similarity of GSU0582 and GSU0935, the results obtained from the potentiometric redox titrations indicate that these sensors are functionally distinct. Unlike GSU0582, the GSU0935 sensor domain displays significant variations in the redox potential: −156 mV for GSU0582 and −251 mV for GSU0935, respectively. The differences observed are most likely caused by the nature of the amino acid residues in the vicinity of the heme groups [145]. The fact that the two sensors have different redox potentials in the same pH range might be physiologically relevant. In fact, it was suggested that the two sensors function in a complementary way to permit the bacteria to adapt to environmental redox potential changes. As depicted in Figure 15, the proposed model suggests that the switch between the oxidized and reduced forms of the sensors—and the concomitant switch of the heme axial ligands—regulate the bacterial motions in distinct redox potential ranges.

## 5. Conclusions

TCS are abundant multistep signaling pathways that respond to a wide range of stimulus. Several components of the TCS have been structurally and/or functionally characterized. For a large majority of TCS, how physiological signals modulate their action is not precisely understood; however, such studies have provided crucial information regarding the modus operandi of TCS. Most HK and RR share the basic structural and functional properties. However, TCS proteins usually exhibit specificity for intermolecular interactions (dimerization). The modular HK and RR architectures permit significant variations in the arrangement of their domains. The signal-linked interconversion changes usually involve the phosphorylation of residues in specific domains, favoring distinct intra- or intermolecular protein–protein interactions that activate or inhibit a particular cellular response. The arrangement of the domains, and hence the regulatory mechanisms, could differ amongst members of the same subfamily. Thus, a proper prediction of such arrangements and precise mechanism still requires further investigation.

The efforts made up until now to characterize systems such those involved in bacterial chemotaxis have provided a broad understanding of how extracellular signals are transduced and allow cells to trigger proper responses. Contrarily to the signaling domains, which are conserved and always located in the cytoplasmic, the sensory domains present significant variations in their structure and membrane topology. The PAS and PAS-like domains are ubiquitous and sense directly or indirectly a large variety of internal or external stimuli. In the future, it would be not surprising if new ligands are discovered and more PAS and PAS-like domains identified and characterized. 

Signaling systems involved in bacterial chemotaxis are highly specialized TCS that during the evolution have adapted the receptor domains in a way that provided the bacteria a high plasticity for input, output and regulation. Organisms that inhabit challenging habitats control their motility and perhaps other yet unveiled processes, by using these specialized systems. The increasing number of chemosensory systems characterized opens new avenues to the clarification of the signal amplification mechanisms [146]. Despite being at the origin of the MCP designation, methylation does not seem to be the only event responsible for the signal transduction mechanisms. In fact, the interplay between the methylation and demethylation rates is crucial to control the interaction between MCP and downstream kinases and consequently for the regulation of the phosphorylation cascade. Thus, understanding of how chemoreceptors coordinate the cellular responses and how the signals, generated from chemoreceptors, are transmitted should be the focus of future research. 

Heme proteins are involved in many different types of biological processes, including chemotaxis. Examples of proteins involved in this process are the GSU0582 and GSU0935 sensors from *G. sulfurreducens.* These proteins are the first members of a newly described bacterial class of *c*-type heme-based sensors. Following the environmental fluctuations in redox potential, conformation changes observed at the heme moiety of these two sensors match those occurring at the global protein structure, suggesting that signaling at the heme group induces conformational alterations that are transmitted to the other protein domains to complete the signal transduction cycle. The characterization of these two chemotaxis sensors provided additional understanding of bacterial signal transduction mechanisms showing that the sensors operate over large and distinct redox potential ranges by linking changes in their heme spin state/coordination to structural alterations that are transmitted to the cytoplasmatic regulatory domains. One may speculate that the same mechanism may be shared by other *c*-type heme-based sensors in *G. sulfurreducens* and other bacteria. The distinct midpoint reduction potential values of each sensor domain suggest that they are functionally relevant in different redox potential ranges with GSU0935 and GSU0582 operating, respectively at lower and higher redox potentials. These outcomes provided for the first time a justification for the co-existence of two similar MCP in *G. sulfurreducens* and how they are designed to allow the bacteria to activate an adequate cellular response in highly variable subsurface habitats. 

## Figures and Tables

**Table 1:** Classification and function of the middle components of chemotaxis signalling pathways.

Protein	Class	Role in Chemotaxis
MCP	Chemoreceptor	Receptor for chemotaxis stimulus.
CheA	Histidine protein kinase	Autophosphorylates in response to MCP. Phosphorylates CheY and CheB.
CheW	Adaptor protein	Involved in the formation of the MCP-CheA-CheW complex.
CheY	Response regulator	Binds FliM when phosphorylated to alter flagellar rotation. Multiple CheY may act as phosphate sinks.
CheB	Response regulator, methylesterase	Removes methyl groups from MCP. Activated upon phosphorylation.
CheR	Methyltransferase	Constitutively methylates MCP.
CheZ	Phosphatase	Stimulates dephosphorylation of CheY and signal termination.
CheV	CheW-CheY fusion	May function as a phosphate sink and signal termination. Alternatively, may function in adaptation.
FliM	Motor switch protein	Alters flagellar rotation in response to CheY~P binding.

**Table 2:** Number of chemotaxis and *che* genes obtained from the analysis of *Geobacter* available genomes.

Strain	Chemotaxis Genes	MCP	*cheA*	*cheB*	*cheR*	*cheW*	*cheY*	*cheC*	*cheD*	*cheX*	*cheV*
*G. sulfurreducens PCA*	79	35	4	4	4	10	10	1	3	4	1
*G. sulfurreducens* *KN400*	77	34	4	4	4	8	11	1	3	4	1
*G. metallireducens*	68	19	5 ^1^	8 ^2^	8 ^2^	8	11 ^1^	1	3	3	1
*G. uraniireducens*	73	27	7	6	9	10	6	1	2	1	1
*G. lovleyi*	68	29	6	5 ^2^	7 ^2^	7	7	2	1	1	2
*G. bemidjiensis*	90	35	8	7	8	11	11	1	3	3	1
*G. daltonii* FRC-32	75	27	7	8 ^2^	11 ^2^	10	6	-	1	1	1
M21	94	37	9	7	10	12	7	2	3	3	1
M18	97	32	10	10	11	14	9	2	2	3	1
*G. pickeringii*	70	18	5	2	6	7	25	-	3	3	1
*G. anodireducens*	57	28	4	1	5	10	-	1	3	4	1
*G. bremensis*	109	38	19	9 ^2^	12 ^2^	1	8	1	3	3	1
*G. soli*	85 ^3^	-	-	-	-	-	-	-	-	-	-

^1^ The number of *cheA* and *cheY* genes includes a contribution from *cheAY* fusion. ^2^ The number of *cheB* and *cheR* genes includes a contribution from *cheBR* fusion. ^3^ The number of chemotaxis genes were described in [138].

## References

[1] Stock A.M., Robinson V.L., Goudreau P.N. (2000). Two-component signal transduction. Annu. Rev. Biochem..

[2] Mizuno T. (1997). Compilation of all genes encoding two-component phosphotransfer signal transducers in the genome of *Escherichia coli*. DNA Res..

[3] Parkinson J.S., Kofoid E.C. (1992). Communication modules in bacterial signaling proteins. Annu. Rev. Genet..

[4] Hoch J.A. (2000). Two-component and phosphorelay signal transduction. Curr. Opin. Microbiol..

[5] Wurgler-Murphy S.M., Saito H. (1997). Two-component signal transducers and MAPK cascades. Trends Biochem. Sci..

[6] Li S., Ault A., Malone C.L., Raitt D., Dean S., Johnston L.H., Deschenes R.J., Fassler J.S. (1998). The yeast histidine protein kinase, Sln1p, mediates phosphotransfer to two response regulators, Ssk1p and Skn7p. EMBO J..

[7] Srikantha T., Tsai L., Daniels K., Enger L., Highley K., Soll D.R. (1998). The two-component hybrid kinase regulator CaNIK1 of *Candida albicans*. Microbiology.

[8] Calera J.A., Zhao X.J., Calderone R. (2000). Defective hyphal development and avirulence caused by a deletion of the SSK1 response regulator gene in *Candida albicans*. Infect. Immun..

[9] Thomason P., Traynor D., Kay R. (1999). Taking the plunge. Terminal differentiation in Dictyostelium. Trends Genet..

[10] Urao T., Yamaguchi-Shinozaki K., Shinozaki K. (2000). Two-component systems in plant signal transduction. Trends Plant. Sci..

[11] Capra E.J., Laub M.T. (2012). Evolution of two-component signal transduction systems. Annu. Rev. Microbiol..

[12] Wolanin P.M., Thomason P.A., Stock J.B. (2002). Histidine protein kinases: Key signal transducers outside the animal kingdom. Genome Biol..

[13] Gislason A.S., Choy M., Bloodworth R.A., Qu W., Stietz M.S., Li X., Zhang C., Cardona S.T. (2017). Competitive Growth Enhances Conditional Growth Mutant Sensitivity to Antibiotics and Exposes a Two-Component System as an Emerging Antibacterial Target in *Burkholderia cenocepacia*. Antimicrob. Agents Chemother..

[14] West A.H., Stock A.M. (2001). Histidine kinases and response regulator proteins in two-component signaling systems. Trends Biochem. Sci..

[15] Groisman E.A. (2016). Feedback Control of Two-Component Regulatory Systems. Annu. Rev. Microbiol..

[16] Bijlsma J.J., Groisman E.A. (2003). Making informed decisions: Regulatory interactions between two-component systems. Trends Microbiol..

[17] Cheung J., Hendrickson W.A. (2010). Sensor domains of two-component regulatory systems. Curr. Opin. Microbiol..

[18] Miller M.B., Skorupski K., Lenz D.H., Taylor R.K., Bassler B.L. (2002). Parallel quorum sensing systems converge to regulate virulence in *Vibrio cholerae*. Cell.

[19] Jiang M., Shao W., Perego M., Hoch J.A. (2000). Multiple histidine kinases regulate entry into stationary phase and sporulation in *Bacillus subtilis*. Mol. Microbiol..

[20] Paul R., Weiser S., Amiot N.C., Chan C., Schirmer T., Giese B., Jenal U. (2004). Cell cycle-dependent dynamic localization of a bacterial response regulator with a novel di-guanylate cyclase output domain. Genes Dev..

[21] Falke J.J., Bass R.B., Butler S.L., Chervitz S.A., Danielson M.A. (1997). The two-component signaling pathway of bacterial chemotaxis: A molecular view of signal transduction by receptors, kinases, and adaptation enzymes. Annu. Rev. Cell Dev. Biol..

[22] Iuchi S., Weiner L. (1996). Cellular and molecular physiology of *Escherichia coli* in the adaptation to aerobic environments. J. Biochem..

[23] Unden G., Bongaerts J. (1997). Alternative respiratory pathways of *Escherichia coli*: Energetics and transcriptional regulation in response to electron acceptors. Biochim. Biophys. Acta.

[24] Hoch J.A. (1993). Regulation of the phosphorelay and the initiation of sporulation in *Bacillus subtilis*. Annu. Rev. Microbiol..

[25] Perego M. (1998). Kinase-phosphatase competition regulates *Bacillus subtilis* development. Trends Microbiol..

[26] Domian I.J., Quon K.C., Shapiro L. (1996). The control of temporal and spatial organization during the Caulobacter cell cycle. Curr. Opin. Genet. Dev..

[27] Wu J., Newton A. (1997). Regulation of the Caulobacter flagellar gene hierarchy; not just for motility. Mol. Microbiol..

[28] Kaplan H.B., Plamann L. (1996). A *Myxococcus xanthus* cell density-sensing system required for multicellular development. FEMS Microbiol. Lett..

[29] Ward M.J., Zusman D.R. (1997). Regulation of directed motility in *Myxococcus xanthus*. Mol. Microbiol..

[30] Krell T., Lacal J., Busch A., Silva-Jimenez H., Guazzaroni M.E., Ramos J.L. (2010). Bacterial sensor kinases: Diversity in the recognition of environmental signals. Annu. Rev. Microbiol..

[31] Taylor B.L., Zhulin I.B. (1999). PAS domains: Internal sensors of oxygen, redox potential, and light. Microbiol. Mol. Biol. Rev..

[32] Finn R.D., Mistry J., Tate J., Coggill P., Heger A., Pollington J.E., Gavin O.L., Gunasekaran P., Ceric G., Forslund K. (2010). The Pfam protein families database. Nucleic Acids Res..

[33] Hefti M.H., Francoijs K.J., de Vries S.C., Dixon R., Vervoort J. (2004). The PAS fold. A redefinition of the PAS domain based upon structural prediction. Eur. J. Biochem..

[34] Scheu P.D., Kim O.B., Griesinger C., Unden G. (2010). Sensing by the membrane-bound sensor kinase DcuS: Exogenous versus endogenous sensing of C(4)-dicarboxylates in bacteria. Future Microbiol..

[35] Upadhyay A.A., Fleetwood A.D., Adebali O., Finn R.D., Zhulin I.B. (2016). Cache Domains That are Homologous to, but Different from PAS Domains Comprise the Largest Superfamily of Extracellular Sensors in Prokaryotes. PLoS Comput. Biol..

[36] Chang C., Tesar C., Gu M., Babnigg G., Joachimiak A., Pokkuluri P.R., Szurmant H., Schiffer M. (2010). Extracytoplasmic PAS-like domains are common in signal transduction proteins. J. Bacteriol..

[37] Moglich A., Ayers R.A., Moffat K. (2009). Structure and signaling mechanism of Per-ARNT-Sim domains. Structure.

[38] Goddard T.D., Huang C.C., Ferrin T.E. (2007). Visualizing density maps with UCSF Chimera. J. Struct. Biol..

[39] Ho Y.S., Burden L.M., Hurley J.H. (2000). Structure of the GAF domain, a ubiquitous signaling motif and a new class of cyclic GMP receptor. EMBO J..

[40] Yang X., Kuk J., Moffat K. (2008). Crystal structure of *Pseudomonas aeruginosa* bacteriophytochrome: Photoconversion and signal transduction. Proc. Natl. Acad. Sci. USA.

[41] Yang X., Kuk J., Moffat K. (2009). Conformational differences between the Pfr and Pr states in *Pseudomonas aeruginosa* bacteriophytochrome. Proc. Natl. Acad. Sci. USA.

[42] Wagner J.R., Zhang J., Brunzelle J.S., Vierstra R.D., Forest K.T. (2007). High resolution structure of Deinococcus bacteriophytochrome yields new insights into phytochrome architecture and evolution. J. Biol. Chem..

[43] Yang X., Stojkovic E.A., Kuk J., Moffat K. (2007). Crystal structure of the chromophore binding domain of an unusual bacteriophytochrome, RpBphP3, reveals residues that modulate photoconversion. Proc. Natl. Acad. Sci. USA.

[44] Essen L.-O., Mailliet J., Hughes J. (2008). The structure of a complete phytochrome sensory module in the Pr ground state. Proc. Natl. Acad. Sci. USA.

[45] Albanesi D., Martin M., Trajtenberg F., Mansilla M.C., Haouz A., Alzari P.M., de Mendoza D., Buschiazzo A. (2009). Structural plasticity and catalysis regulation of a thermosensor histidine kinase. Proc. Natl. Acad. Sci. USA.

[46] Martin M., Albanesi D., Alzari P.M., de Mendoza D. (2009). Functional in vitro assembly of the integral membrane bacterial thermosensor DesK. Protein Expr. Purif..

[47] Bogel G., Schrempf H., de Orue Lucana D.O. (2009). The heme-binding protein HbpS regulates the activity of the *Streptomyces reticuli* iron-sensing histidine kinase SenS in a redox-dependent manner. Amino Acids.

[48] Voet-van-Vormizeele J., Groth G. (2008). Ethylene controls autophosphorylation of the histidine kinase domain in ethylene receptor ETR1. Mol. Plant.

[49] Geisinger E., George E.A., Chen J., Muir T.W., Novick R.P. (2008). Identification of ligand specificity determinants in AgrC, the *Staphylococcus aureus* quorum-sensing receptor. J. Biol. Chem..

[50] Jensen R.O., Winzer K., Clarke S.R., Chan W.C., Williams P. (2008). Differential recognition of *Staphylococcus aureus* quorum-sensing signals depends on both extracellular loops 1 and 2 of the transmembrane sensor AgrC. J. Mol. Biol..

[51] Gordeliy V.I., Labahn J., Moukhametzianov R., Efremov R., Granzin J., Schlesinger R., Buldt G., Savopol T., Scheidig A.J., Klare J.P. (2002). Molecular basis of transmembrane signalling by sensory rhodopsin II-transducer complex. Nature.

[52] Lacal J., Garcia-Fontana C., Munoz-Martinez F., Ramos J.L., Krell T. (2010). Sensing of environmental signals: Classification of chemoreceptors according to the size of their ligand binding regions. Environ. Microbiol..

[53] Cheung J., Hendrickson W.A. (2008). Crystal structures of C4-dicarboxylate ligand complexes with sensor domains of histidine kinases DcuS and DctB. J. Biol. Chem..

[54] Gao R., Stock A.M. (2009). Biological insights from structures of two-component proteins. Annu. Rev. Microbiol..

[55] Shu C.J., Ulrich L.E., Zhulin I.B. (2003). The NIT domain: A predicted nitrate-responsive module in bacterial sensory receptors. Trends Biochem. Sci..

[56] Emami K., Topakas E., Nagy T., Henshaw J., Jackson K.A., Nelson K.E., Mongodin E.F., Murray J.W., Lewis R.J., Gilbert H.J. (2009). Regulation of the xylan-degrading apparatus of *Cellvibrio japonicus* by a novel two-component system. J. Biol. Chem..

[57] Zhou Y.F., Nan B., Nan J., Ma Q., Panjikar S., Liang Y.H., Wang Y., Su X.D. (2008). C4-dicarboxylates sensing mechanism revealed by the crystal structures of DctB sensor domain. J. Mol. Biol..

[58] Yeh J.I., Biemann H.P., Prive G.G., Pandit J., Koshland D.E., Kim S.H. (1996). High-resolution structures of the ligand binding domain of the wild-type bacterial aspartate receptor. J. Mol. Biol..

[59] Anantharaman V., Aravind L. (2000). Cache—A signaling domain common to animal Ca(2+)-channel subunits and a class of prokaryotic chemotaxis receptors. Trends Biochem. Sci..

[60] Girvan H.M., Munro A.W. (2013). Heme sensor proteins. J. Biol. Chem..

[61] Farhana A., Saini V., Kumar A., Lancaster J.R., Steyn A.J. (2012). Environmental heme-based sensor proteins: Implications for understanding bacterial pathogenesis. Antioxid. Redox. Signal..

[62] Gilles-Gonzalez M.A., Gonzalez G. (2004). Signal transduction by heme-containing PAS-domain proteins. J. Appl. Physiol..

[63] Gilles-Gonzalez M.A., Gonzalez G., Perutz M.F., Kiger L., Marden M.C., Poyart C. (1994). Heme-based sensors, exemplified by the kinase FixL, are a new class of heme protein with distinctive ligand binding and autoxidation. Biochemistry.

[64] Tomita T., Gonzalez G., Chang A.L., Ikeda-Saito M., Gilles-Gonzalez M.A. (2002). A comparative resonance Raman analysis of heme-binding PAS domains: Heme iron coordination structures of the BjFixL, AxPDEA1, EcDos, and MtDos proteins. Biochemistry.

[65] Gilles-Gonzalez M.A., Gonzalez G. (2005). Heme-based sensors: Defining characteristics, recent developments, and regulatory hypotheses. J. Inorg. Biochem..

[66] Crosson S., McGrath P.T., Stephens C., McAdams H.H., Shapiro L. (2005). Conserved modular design of an oxygen sensory/signaling network with species-specific output. Proc. Natl. Acad. Sci. USA.

[67] David M., Daveran M.L., Batut J., Dedieu A., Domergue O., Ghai J., Hertig C., Boistard P., Kahn D. (1988). Cascade regulation of nif gene expression in *Rhizobium meliloti*. Cell.

[68] Rey F.E., Harwood C.S. (2010). FixK, a global regulator of microaerobic growth, controls photosynthesis in *Rhodopseudomonas palustris*. Mol. Microbiol..

[69] Gilles-Gonzalez M.A., Ditta G.S., Helinski D.R. (1991). A haemoprotein with kinase activity encoded by the oxygen sensor of *Rhizobium meliloti*. Nature.

[70] Delgado-Nixon V.M., Gonzalez G., Gilles-Gonzalez M.A. (2000). Dos, a heme-binding PAS protein from *Escherichia coli*, is a direct oxygen sensor. Biochemistry.

[71] Tanaka A., Takahashi H., Shimizu T. (2007). Critical role of the heme axial ligand, Met95, in locking catalysis of the phosphodiesterase from *Escherichia coli* (Ec DOS) toward Cyclic diGMP. J. Biol. Chem..

[72] Tuckerman J.R., Gonzalez G., Sousa E.H., Wan X., Saito J.A., Alam M., Gilles-Gonzalez M.A. (2009). An oxygen-sensing diguanylate cyclase and phosphodiesterase couple for c-di-GMP control. Biochemistry.

[73] Cho H.Y., Cho H.J., Kim Y.M., Oh J.I., Kang B.S. (2009). Structural insight into the heme-based redox sensing by DosS from *Mycobacterium tuberculosis*. J. Biol. Chem..

[74] Podust L.M., Ioanoviciu A., Ortiz de Montellano P.R. (2008). 2.3 A X-ray structure of the heme-bound GAF domain of sensory histidine kinase DosT of *Mycobacterium tuberculosis*. Biochemistry.

[75] Molitor B., Stassen M., Modi A., El-Mashtoly S.F., Laurich C., Lubitz W., Dawson J.H., Rother M., Frankenberg-Dinkel N. (2013). A heme-based redox sensor in the methanogenic archaeon *Methanosarcina acetivorans*. J. Biol. Chem..

[76] Motomura T., Suga M., Hienerwadel R., Nakagawa A., Lai T.L., Nitschke W., Kuma T., Sugiura M., Boussac A., Shen J.R. (2017). Crystal structure and redox properties of a novel cyanobacterial heme protein with a His/Cys heme axial ligation and a Per-Arnt-Sim (PAS)-like domain. J. Biol. Chem..

[77] Yoshioka S., Kobayashi K., Yoshimura H., Uchida T., Kitagawa T., Aono S. (2005). Biophysical properties of a *c*-type heme in chemotaxis signal transducer protein DcrA. Biochemistry.

[78] Pokkuluri P.R., Pessanha M., Londer Y.Y., Wood S.J., Duke N.E., Wilton R., Catarino T., Salgueiro C.A., Schiffer M. (2008). Structures and solution properties of two novel periplasmic sensor domains with *c*-type heme from chemotaxis proteins of *Geobacter sulfurreducens*: Implications for signal transduction. J. Mol. Biol..

[79] Londer Y.Y., Dementieva I.S., D’Ausilio C.A., Pokkuluri P.R., Schiffer M. (2006). Characterization of a c-type heme-containing PAS sensor domain from *Geobacter sulfurreducens* representing a novel family of periplasmic sensors in Geobacteraceae and other bacteria. FEMS Microbiol. Lett..

[80] Saito H. (2001). Histidine phosphorylation and two-component signaling in eukaryotic cells. Chem. Rev..

[81] Martinez-Hackert E., Stock A.M. (1997). Structural relationships in the OmpR family of winged-helix transcription factors. J. Mol. Biol..

[82] Milani M., Leoni L., Rampioni G., Zennaro E., Ascenzi P., Bolognesi M. (2005). An active-like structure in the unphosphorylated StyR response regulator suggests a phosphorylation- dependent allosteric activation mechanism. Structure.

[83] Batchelor J.D., Doucleff M., Lee C.J., Matsubara K., De Carlo S., Heideker J., Lamers M.H., Pelton J.G., Wemmer D.E. (2008). Structure and regulatory mechanism of *Aquifex aeolicus* NtrC4: Variability and evolution in bacterial transcriptional regulation. J. Mol. Biol..

[84] Sidote D.J., Barbieri C.M., Wu T., Stock A.M. (2008). Structure of the *Staphylococcus aureus* AgrA LytTR domain bound to DNA reveals a beta fold with an unusual mode of binding. Structure.

[85] O’Hara B.P., Norman R.A., Wan P.T., Roe S.M., Barrett T.E., Drew R.E., Pearl L.H. (1999). Crystal structure and induction mechanism of AmiC-AmiR: A ligand-regulated transcription antitermination complex. EMBO J..

[86] Guhaniyogi J., Wu T., Patel S.S., Stock A.M. (2008). Interaction of CheY with the C-terminal peptide of CheZ. J. Bacteriol..

[87] Okamura H., Hanaoka S., Nagadoi A., Makino K., Nishimura Y. (2000). Structural comparison of the PhoB and OmpR DNA-binding/transactivation domains and the arrangement of PhoB molecules on the phosphate box. J. Mol. Biol..

[88] Wright G.S.A., Saeki A., Hikima T., Nishizono Y., Hisano T., Kamaya M., Nukina K., Nishitani H., Nakamura H., Yamamoto M. (2018). Architecture of the complete oxygen-sensing FixL-FixJ two-component signal transduction system. Sci. Signal..

[89] Boudes M., Lazar N., Graille M., Durand D., Gaidenko T.A., Stewart V., van Tilbeurgh H. (2012). The structure of the NasR transcription antiterminator reveals a one-component system with a NIT nitrate receptor coupled to an ANTAR RNA-binding effector. Mol. Microbiol..

[90] West A.H., Martinez-Hackert E., Stock A.M. (1995). Crystal structure of the catalytic domain of the chemotaxis receptor methylesterase, CheB. J. Mol. Biol..

[91] Tabib-Salazar A., Liu B., Barker D., Burchell L., Qimron U., Matthews S.J., Wigneshweraraj S. (2018). T7 phage factor required for managing RpoS in *Escherichia coli*. Proc. Natl. Acad. Sci. USA.

[92] Sevvana M., Vijayan V., Zweckstetter M., Reinelt S., Madden D.R., Herbst-Irmer R., Sheldrick G.M., Bott M., Griesinger C., Becker S. (2008). A ligand-induced switch in the periplasmic domain of sensor histidine kinase CitA. J. Mol. Biol..

[93] Cheung J., Hendrickson W.A. (2009). Structural analysis of ligand stimulation of the histidine kinase NarX. Structure.

[94] Cheung J., Le-Khac M., Hendrickson W.A. (2009). Crystal structure of a histidine kinase sensor domain with similarity to periplasmic binding proteins. Proteins.

[95] Etzkorn M., Kneuper H., Dunnwald P., Vijayan V., Kramer J., Griesinger C., Becker S., Unden G., Baldus M. (2008). Plasticity of the PAS domain and a potential role for signal transduction in the histidine kinase DcuS. Nat. Struct. Mol. Biol..

[96] Moore J.O., Hendrickson W.A. (2009). Structural analysis of sensor domains from the TMAO-responsive histidine kinase receptor TorS. Structure.

[97] Neiditch M.B., Federle M.J., Pompeani A.J., Kelly R.C., Swem D.L., Jeffrey P.D., Bassler B.L., Hughson F.M. (2006). Ligand-induced asymmetry in histidine sensor kinase complex regulates quorum sensing. Cell.

[98] Ward S.M., Delgado A., Gunsalus R.P., Manson M.D. (2002). A NarX-Tar chimera mediates repellent chemotaxis to nitrate and nitrite. Mol. Microbiol..

[99] Yoshida T., Phadtare S., Inouye M. (2007). The design and development of Tar-EnvZ chimeric receptors. Methods Enzymol..

[100] Cheung J., Bingman C.A., Reyngold M., Hendrickson W.A., Waldburger C.D. (2008). Crystal structure of a functional dimer of the PhoQ sensor domain. J. Biol. Chem..

[101] Goldberg S.D., Soto C.S., Waldburger C.D., Degrado W.F. (2008). Determination of the physiological dimer interface of the PhoQ sensor domain. J. Mol. Biol..

[102] Hulko M., Berndt F., Gruber M., Linder J.U., Truffault V., Schultz A., Martin J., Schultz J.E., Lupas A.N., Coles M. (2006). The HAMP domain structure implies helix rotation in transmembrane signaling. Cell.

[103] Lee J., Tomchick D.R., Brautigam C.A., Machius M., Kort R., Hellingwerf K.J., Gardner K.H. (2008). Changes at the KinA PAS-A dimerization interface influence histidine kinase function. Biochemistry.

[104] Gong W., Hao B., Mansy S.S., Gonzalez G., Gilles-Gonzalez M.A., Chan M.K. (1998). Structure of a biological oxygen sensor: A new mechanism for heme-driven signal transduction. Proc. Natl. Acad. Sci. USA.

[105] Hao B., Isaza C., Arndt J., Soltis M., Chan M.K. (2002). Structure-based mechanism of O_2_ sensing and ligand discrimination by the FixL heme domain of *Bradyrhizobium japonicum*. Biochemistry.

[106] Lee J.M., Cho H.Y., Cho H.J., Ko I.J., Park S.W., Baik H.S., Oh J.H., Eom C.Y., Kim Y.M., Kang B.S. (2008). O_2_- and NO-sensing mechanism through the DevSR two-component system in *Mycobacterium smegmatis*. J. Bacteriol..

[107] Anantharaman V., Balaji S., Aravind L. (2006). The signaling helix: A common functional theme in diverse signaling proteins. Biol. Direct..

[108] Silverman M., Simon M. (1977). Chemotaxis in *Escherichia coli*: Methylation of che gene products. Proc. Natl. Acad. Sci. USA.

[109] Macnab R.M., Koshland D.E. (1972). The gradient-sensing mechanism in bacterial chemotaxis. Proc. Natl. Acad. Sci. USA.

[110] Adler J., Dahl M.M. (1967). A method for measuring the motility of bacteria and for comparing random and non-random motility. J. Gen. Microbiol..

[111] Faguy D.M., Jarrell K.F. (1999). A twisted tale: The origin and evolution of motility and chemotaxis in prokaryotes. Microbiology.

[112] Kort E.N., Goy M.F., Larsen S.H., Adler J. (1975). Methylation of a membrane protein involved in bacterial chemotaxis. Proc. Natl. Acad. Sci. USA.

[113] Bibikov S.I., Biran R., Rudd K.E., Parkinson J.S. (1997). A signal transducer for aerotaxis in *Escherichia coli*. J. Bacteriol..

[114] Rebbapragada A., Johnson M.S., Harding G.P., Zuccarelli A.J., Fletcher H.M., Zhulin I.B., Taylor B.L. (1997). The Aer protein and the serine chemoreceptor Tsr independently sense intracellular energy levels and transduce oxygen, redox, and energy signals for *Escherichia coli* behavior. Proc. Natl. Acad. Sci. USA.

[115] Kleene S.J., Toews M.L., Adler J. (1977). Isolation of glutamic acid methyl ester from an *Escherichia coli* membrane protein involved in chemotaxis. J. Biol. Chem..

[116] Kleene S.J., Hobson A.C., Adler J. (1979). Attractants and repellents influence methylation and demethylation of methyl-accepting chemotaxis proteins in an extract of *Escherichia coli*. Proc. Natl. Acad. Sci. USA.

[117] Alexander R.P., Zhulin I.B. (2007). Evolutionary genomics reveals conserved structural determinants of signaling and adaptation in microbial chemoreceptors. Proc. Natl. Acad. Sci. USA.

[118] Methe B.A., Nelson K.E., Eisen J.A., Paulsen I.T., Nelson W., Heidelberg J.F., Wu D., Wu M., Ward N., Beanan M.J. (2003). Genome of *Geobacter sulfurreducens*: Metal reduction in subsurface environments. Science.

[119] Milburn M.V., Prive G.G., Milligan D.L., Scott W.G., Yeh J., Jancarik J., Koshland D.E., Kim S.H. (1991). Three-dimensional structures of the ligand-binding domain of the bacterial aspartate receptor with and without a ligand. Science.

[120] Tran H.T., Krushkal J., Antommattei F.M., Lovley D.R., Weis R.M. (2008). Comparative genomics of Geobacter chemotaxis genes reveals diverse signaling function. BMC Genom..

[121] Zhulin I.B. (2001). The superfamily of chemotaxis transducers: From physiology to genomics and back. Adv. Microb. Physiol..

[122] Wuichet K., Alexander R.P., Zhulin I.B. (2007). Comparative genomic and protein sequence analyses of a complex system controlling bacterial chemotaxis. Methods Enzymol..

[123] Hazelbauer G.L., Falke J.J., Parkinson J.S. (2008). Bacterial chemoreceptors: High-performance signaling in networked arrays. Trends Biochem. Sci..

[124] Khursigara C.M., Wu X., Zhang P., Lefman J., Subramaniam S. (2008). Role of HAMP domains in chemotaxis signaling by bacterial chemoreceptors. Proc. Natl. Acad. Sci. USA.

[125] Zhulin I.B. (2000). A novel phototaxis receptor hidden in the cyanobacterial genome. J. Mol. Microbiol. Biotechnol..

[126] Wuichet K., Zhulin I.B. (2003). Molecular evolution of sensory domains in cyanobacterial chemoreceptors. Trends Microbiol..

[127] Anand G.S., Goudreau P.N., Stock A.M. (1998). Activation of methylesterase CheB: Evidence of a dual role for the regulatory domain. Biochemistry.

[128] Hess J.F., Oosawa K., Kaplan N., Simon M.I. (1988). Phosphorylation of three proteins in the signaling pathway of bacterial chemotaxis. Cell.

[129] Kato J., Nakamura T., Kuroda A., Ohtake H. (1999). Cloning and characterization of chemotaxis genes in *Pseudomonas aeruginosa*. Biosci. Biotechnol. Biochem..

[130] Manson M.D. (2008). The tie that binds the dynamic duo: The connector between AS1 and AS2 in the HAMP domain of the *Escherichia coli* Tsr chemoreceptor. J. Bacteriol..

[131] Welch M., Oosawa K., Aizawa S., Eisenbach M. (1993). Phosphorylation-dependent binding of a signal molecule to the flagellar switch of bacteria. Proc. Natl. Acad. Sci. USA.

[132] Toker A.S., Macnab R.M. (1997). Distinct regions of bacterial flagellar switch protein FliM interact with FliG, FliN and CheY. J. Mol. Biol..

[133] McEvoy M.M., Bren A., Eisenbach M., Dahlquist F.W. (1999). Identification of the binding interfaces on CheY for two of its targets, the phosphatase CheZ and the flagellar switch protein fliM. J. Mol. Biol..

[134] Lovley D.R., Holmes D.E., Nevin K.P. (2004). Dissimilatory Fe(III) and Mn(IV) reduction. Adv. Microb. Physiol..

[135] Nevin K.P., Kim B.C., Glaven R.H., Johnson J.P., Woodard T.L., Methe B.A., Didonato R.J., Covalla S.F., Franks A.E., Liu A. (2009). Anode biofilm transcriptomics reveals outer surface components essential for high density current production in *Geobacter sulfurreducens* fuel cells. PLoS ONE.

[136] Lovley D.R. (2006). Bug juice: Harvesting electricity with microorganisms. Nat. Rev. Microbiol..

[137] Lovley D.R. (2003). Cleaning up with genomics: Applying molecular biology to bioremediation. Nat. Rev. Microbiol..

[138] Yang G., Chen S., Zhou S., Liu Y. (2015). Genome sequence of a dissimilatory Fe(III)-reducing bacterium *Geobacter soli* type strain GSS01(T). Stand. Genom. Sci..

[139] Coppi M.V., Leang C., Sandler S.J., Lovley D.R. (2001). Development of a genetic system for *Geobacter sulfurreducens*. Appl. Environ. Microbiol..

[140] Butler J.E., Young N.D., Aklujkar M., Lovley D.R. (2012). Comparative genomic analysis of *Geobacter sulfurreducens* KN400, a strain with enhanced capacity for extracellular electron transfer and electricity production. BMC Genom..

[141] Aklujkar M., Krushkal J., DiBartolo G., Lapidus A., Land M.L., Lovley D.R. (2009). The genome sequence of *Geobacter metallireducens*: Features of metabolism, physiology and regulation common and dissimilar to *Geobacter sulfurreducens*. BMC Microbiol..

[142] Altschul S.F., Madden T.L., Schaffer A.A., Zhang J., Zhang Z., Miller W., Lipman D.J. (1997). Gapped BLAST and PSI-BLAST: A new generation of protein database search programs. Nucleic Acids Res..

[143] Kelley L.A., Sternberg M.J. (2009). Protein structure prediction on the Web: A case study using the Phyre server. Nat. Protoc.

[144] Catarino T., Pessanha M., De Candia A.G., Gouveia Z., Fernandes A.P., Pokkuluri P.R., Murgida D., Marti M.A., Todorovic S., Salgueiro C.A. (2010). Probing the chemotaxis periplasmic sensor domains from *Geobacter sulfurreducens* by combined resonance Raman and molecular dynamic approaches: NO and CO sensing. J. Phys. Chem. B.

[145] Mao J., Hauser K., Gunner M.R. (2003). How cytochromes with different folds control heme redox potentials. Biochemistry.

[146] Sourjik V., Berg H.C. (2004). Functional interactions between receptors in bacterial chemotaxis. Nature.

